# Exploring the secrets of marine microorganisms: Unveiling secondary metabolites through metagenomics

**DOI:** 10.1111/1751-7915.14533

**Published:** 2024-07-29

**Authors:** Shaoyu Wang, Xinyan Li, Weiqin Yang, Ranran Huang

**Affiliations:** ^1^ Institute of Marine Science and Technology Shandong University Qingdao Shandong China; ^2^ Qingdao Key Laboratory of Ocean Carbon Sequestration and Negative Emission Technology Shandong University Qingdao China; ^3^ School of Computer Science and Technology Shandong University Qingdao Shandong China; ^4^ Global Ocean Negative Carbon Emissions (ONCE) Program Alliance Qingdao China

## Abstract

Marine microorganisms are increasingly recognized as primary producers of marine secondary metabolites, drawing growing research interest. Many of these organisms are unculturable, posing challenges for study. Metagenomic techniques enable research on these unculturable microorganisms, identifying various biosynthetic gene clusters (BGCs) related to marine microbial secondary metabolites, thereby unveiling their secrets. This review comprehensively analyses metagenomic methods used in discovering marine microbial secondary metabolites, highlighting tools commonly employed in BGC identification, and discussing the potential and challenges in this field. It emphasizes the key role of metagenomics in unveiling secondary metabolites, particularly in marine sponges and tunicates. The review also explores current limitations in studying these metabolites through metagenomics, noting how long‐read sequencing technologies and the evolution of computational biology tools offer more possibilities for BGC discovery. Furthermore, the development of synthetic biology allows experimental validation of computationally identified BGCs, showcasing the vast potential of metagenomics in mining marine microbial secondary metabolites.

## Graphical abstract

Marine microorganisms are primary producers of secondary metabolites, often originating from unculturable microorganisms, posing significant research challenges. This review explores metagenomic techniques for identifying biosynthetic gene clusters (BGCs) associated with these metabolites, emphasizing the role of metagenomics in sponges and tunicates. The article also discusses current limitations and the potential of artificial intelligence, long‐read sequencing, and synthetic biology in advancing BGC discovery and the practical application of secondary metabolites.

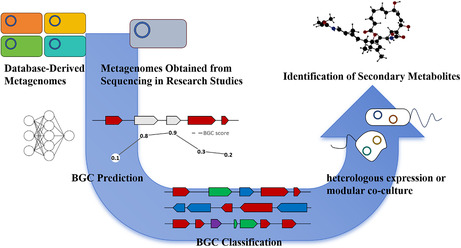

## INTRODUCTION

The ocean, Earth's largest ecosystem, presents a complex ecological environment distinct from terrestrial ecosystems, characterized by high pressure, high salinity, and limited light (Nigam et al., 2019; Reuver et al., 2022). This unique marine setting necessitates specific adaptations in marine organisms, leading to distinctive metabolic patterns. Such distinctiveness harbours extensive potential for discovering new secondary metabolites (Bhatnagar & Kim, 2010; David et al., 2015; Jensen et al., 2015; Mehbub et al., 2014; Nigam et al., 2019; Subramani & Aalbersberg, 2013; Xiliang et al., 2021; Zhang et al., 2005). Marine organisms, which are home to an estimated 10 million species, constitute a significant portion of the Earth's biomass. These organisms account for approximately 87% of the total carbon mass on the planet, highlighting their crucial role in the global carbon cycle and their substantial contribution to the Earth's overall biomass (Bar‐On et al., 2018; Wang, 2016). Research in this area has already identified potential anticancer drugs and other bioactive substances in marine organisms, marking significant progress in medicine and biotechnology (Khalifa et al., 2019; Martins et al., 2014).

Marine microorganisms, constituting about 70% of the ocean's biomass, are an essential component of marine life (Bar‐On et al., 2018). The intricate marine environment has driven the evolution of various unique metabolic pathways in these organisms. Traditional experimental methods, however, face limitations in comprehending the immense diversity of marine microorganisms. A vast number of undiscovered and uncultivable microbes remain in the ocean, posing significant challenges to revealing their secrets (Marmann et al., 2014; Romano et al., 2018).

Since its inception in 1998, metagenomics has undergone rapid development over the past 33 years. Initially focused on uncultivated soil microorganisms (Handelsman et al., 1998), the field, propelled by advances in sequencing technologies and bioinformatics, has broadened to encompass various disciplines (Breitwieser & Salzberg, 2019; Chiu & Miller, 2019; Gu et al., 2020; Key et al., 2017; Ko et al., 2022). This evolution in technology has significantly advanced the study of uncultivable marine microorganisms and opened up new avenues for exploring marine microbial life and their metabolites. The rapid progress of metagenomics provides an effective tool and framework for deepening our understanding of marine microorganisms and microbial communities across different ecosystems (Alma'abadi et al., 2015; Zhang et al., 2021). Such advancements offer scientists novel opportunities to expand the discovery of natural products, thereby playing a crucial role in uncovering unknown facets of marine microbiology (Coutinho et al., 2018; Kodzius & Gojobori, 2015).

Investigating the secondary metabolites of marine microorganisms offer insights into their chemical communication, defence mechanisms, inter‐microbial relationships, and food chain dynamics in marine ecosystems (Monti, 2023; Suryanarayanan, 2012; Teasdale et al., 2009). Viewing bioactive substances through an ecological lens provides a macroscopic perspective on the role of these compounds, foundational for marine chemical ecology research (Hay, 1996). Additionally, studies suggest that the count of biosynthetic gene clusters (BGCs) involved in producing secondary metabolites in microorganisms significantly exceeds the number of identified secondary metabolites (Hoshino et al., 2019; Knowles et al., 2022; Marmann et al., 2014; Zhuang & Zhang, 2021). Therefore, utilizing metagenomics to explore unexpressed or low‐expressed BGCs is a crucial approach for discovering structurally novel and bioactive natural products from microorganisms Consequently, employing metagenomics to examine unexpressed or low‐expressed BGCs emerges as a key strategy for discovering structurally unique and bioactive natural products from microorganisms (Sekurova et al., 2019; Zhou et al., 2021).

### Multi‐layered technological strategies in the study of novel secondary metabolites from marine microorganisms

#### BGC databases

As research into secondary metabolites deepens, databases cataloguing various secondary metabolites have been established. Currently, the analysis and comparison of BGCs primarily rely on these BGC databases. In 2013, Conway and Boddy (2012)founded the initial known product BGC database, ClusterMine360, now encompassing over 200 clusters from 185 compound families. This database also features a distinctive sequence library with over 10,000 polyketide synthase/non‐ribosomal peptide synthetase structural domains (Conway & Boddy, 2012).

In 2015, Medema et al. (2015) launched the Minimum Information about a Biosynthetic Gene cluster (MIBiG) data standard, initially comprising 1170 BGC entries. MIBiG was updated to version 2.0 in 2019, incorporating 851 additional entries compared to its initial version (Kautsar et al., 2020). In 2022, the database progressed to version 3.0, adding 661 new BGCs and removing 69 entries due to low quality or redundancy (Terlouw et al., 2023). MIBiG is now extensively used in developing and evaluating various BGC prediction programmes.

With the advancement of computational biology, databases containing predicted BGCs derived from various software tools have also been compiled, complementing those based on actual products. In 2021, Kautsar, Blin, et al. (2021),Kautsar, van der Hooft, et al. (2021) created BiG‐FAM, which includes predictions from 1,225,071 BGCs sourced from 209,206 publicly available microbial genomes and metagenome‐assembled genomes. Such databases of BGCs are invaluable as foundational resources for discovering new secondary metabolites.

#### Computational biology approach to mining more BGCs

With the ongoing advancement in secondary metabolite research, an increasing number of these compounds and their BGCs have been identified. As data accumulates, the application of artificial intelligence (AI) methods to the study of secondary metabolites has become increasingly feasible. This advancement allows AI technology to gradually permeate secondary metabolite research, offering more possibilities and new research pathways.

antiSMASH, introduced in 2011, has rapidly evolved into the most widely utilized tool for detecting and identifying BGCs in archaea, bacteria, and fungi (Blin et al., 2023). ClusterFinder was developed in 2014 and based on a dual‐state Hidden Markov Model (HMM) (Cimermancic et al., 2014). DeepBGC, launched in 2019, employed a detailed process, including gene ORF detection, protein domain analysis, domain‐level and protein‐level BGC detection, post‐processing, and classification. It set a new standard using the MiBIG 1.3 database, integrating advanced methods like BiLSTM and RF (Hannigan et al., 2019).

Machine learning‐based programmes for predicting secondary metabolites have found extensive application across diverse research domains (Yuan et al., 2023). The ongoing development and enhancement of these tools equip researchers with efficient, precise, and interpretable methods, fuelling further progress in the exploration of secondary metabolites.

#### Cluster analysis of BGC

For the effective identification of numerous BGCs from metagenomic datasets using computational biology, selecting a subset of representative and promising BGCs for further analysis is a more feasible approach. Recent studies have begun utilizing computational methods to group similar BGCs, using known BGCs of the same type to predict potential products, functions, and evolutionary histories of newly identified BGCs (Doroghazi et al., 2014; Duncan et al., 2015). This strategy may also reveal entirely new BGC types, offering directions for discovering novel secondary metabolites (Cimermancic et al., 2014; Nielsen et al., 2017). To facilitate this, Navarro‐Muñoz et al. in Navarro‐Muñoz et al., 2020 developed a computational workflow that integrates the “biosynthetic gene similarity clustering and prospecting engine” (BiG‐SCAPE) and “core analysis of syntenic orthologues to prioritize natural product gene clusters” (CORASON). This workflow rapidly groups BGCs into gene cluster families (GCFs) and further categorizes them into gene cluster clans (Navarro‐Muñoz et al., 2020). In 2021, BiG‐SLiCE was introduced, significantly reducing the computational resources needed for grouping BGCs into GCFs and broadening the method's applicability (Kautsar, Blin, et al., 2021; Kautsar, van der Hooft, et al., 2021).

In summary, the evolution of BGC databases and computational biology has significantly propelled the study of secondary metabolites. These advancements have not only streamlined the identification and analysis of BGSs but also opened new avenues for discovering novel compounds. By leveraging advanced computational tools and methodologies, researchers can now delve deeper into the complex world of secondary metabolites, paving the way for ground‐breaking discoveries in marine microbiology and beyond.

### Metagenomics reveals novel secondary metabolites from marine microorganisms

**FIGURE 1:**
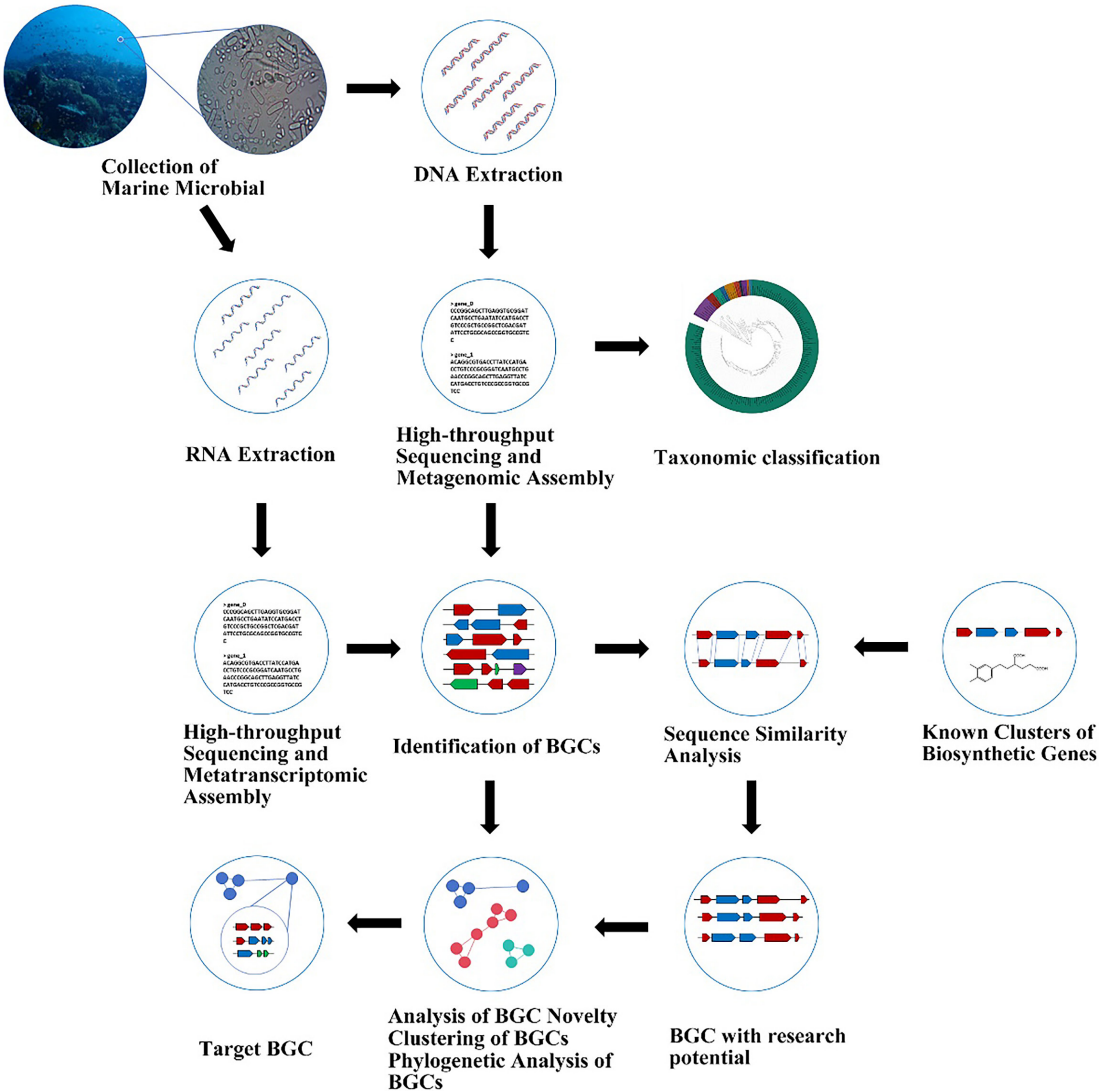
Workflow for identifying and analysing marine microbial BGCs collection: Marine microbial samples are collected. DNA/RNA Extraction: DNA and RNA are extracted from samples. Sequencing and Assembly: High‐throughput sequencing and assembly of metagenomic and metatranscriptomic data. Taxonomic Classification: Classification of species present in the samples. BGC Identification: Detection of BGCs from the assembled data. Sequence similarity analysis: Comparison of BGCs with known biosynthetic gene clusters. Known clusters: Identification of similarities with known BGCs. Research potential: identification of BGCs with novel research potential. Novelty and phylogenetic analysis: analysis of BGC novelty, clustering, and phylogenetic relationships.

Metagenomics technology offers a ground‐breaking perspective in understanding the potential of new secondary metabolites from marine microorganisms, given the vastness of the oceans (Figure 1). Paoli and colleagues have demonstrated the significant exploration potential of these metabolites across the entire ocean. They compiled approximately 10,000 microbial genomes from cultured bacteria and single cells, and over 25,000 reconstructed metagenomes from more than 1000 seawater samples. Employing antiSMASH, they identified about 40,000 potential novel BGCs and discovered bacteria in the Ca. Eudoremicrobiaceae group, noted for their robust secondary metabolite synthesis capabilities (Paoli et al., 2022).

In their study, focusing on specific oceanic regions, Rego and colleagues assessed the diversity of metagenomic biosynthesis in the Arctic Ocean. They successfully recovered 149 ketosynthase (KS) domain operational taxonomic unit sequences, with 36% being unassignable to any known BGC. Furthermore, from 74 bacterial metagenome‐assembled genomes, they extracted 179 BGCs, underscoring the Arctic Ocean's potential as a source of novel secondary metabolites (Rego et al., 2021).

In the Northwest Pacific, the study by Wang et al., 2023 identified 1195 BGCs in metagenomes obtained from surface seawater, with numerous terpene synthase clusters and Pfam domains for squalene/phytoene synthesis demonstrating the microbial biosynthetic potential of the region.

In the deep‐sea sediments of the Gulf of Mexico, Fernández‐López and their team investigated the metagenome for type I polyketide synthase (PKS) genes. They identified more than 2000 candidate genes encoding type I PKS domains, as well as potential biosynthetic pathways for various other secondary metabolites (Fernández‐López et al., 2022).

Cuadrat and colleagues performed a metagenomic analysis on seawater influenced by upwelling off the Brazilian coast, resulting in the identification of 84 KS and 46 cyclization (C) domain sequences. This particular environment demonstrated significant potential for discovering novel secondary metabolites of biotechnological value (Cuadrat et al., 2015).

These metagenomic studies collectively highlight the vast potential for development across the entire ocean. The outcomes lay a solid groundwork for ongoing research into secondary metabolites derived from marine microorganisms. A substantial number of potential BGCs have been catalogued in databases, offering invaluable references for future studies and facilitating mutual validation.

### The application of metagenomics Technology in the Exploration of secondary metabolites within marine symbiotic systems

Symbiotic systems have emerged as a key area in the quest for novel secondary metabolites. Numerous studies highlight that these metabolites are prevalent within symbiotic systems, playing essential roles such as defence, communication, and regulation (Engel et al., 2015; Kaasalainen et al., 2012; Sagar et al., 2010). These secondary metabolites are not only vital for the survival of the organisms involved but also serve as a valuable source of biological resources for human exploration. However, culturing microorganisms from symbiotic systems in pure form poses significant challenges. Often, these organisms do not express crucial products in isolation (Bertrand et al., 2014; Woodhouse et al., 2013), and co‐cultivation presents its own difficulties (Lewis et al., 2021). Utilizing metagenomics technology, researchers can circumvent these cultivation challenges, directly investigating symbiotic systems with greater efficiency (Arumugam et al., 2011; Grube et al., 2015; Ni & Tokuda, 2013). Presently, extensive research focuses on using metagenomic methods to explore oceanic symbiotic systems, with the goal of deepening the mining and understanding of secondary metabolites.

### Marine sponge symbiotic systems

Marine sponges and their microbial symbiotic communities are recognized as prolific sources of diverse natural products (Taylor et al., 2007). In certain sponge species, microbes can comprise 40%–60% of the sponge's tissue volume (Fathima et al., 2022; Yang, Franco, & Zhang, 2019, Yang, Qian, et al., 2019). Extensive research has shown that these microbes, especially sponge‐associated actinomycetes, are the primary producers of structurally varied and pharmacologically promising secondary metabolites (Jensen, 2016; Thomas et al., 2010; Yang, Franco, & Zhang, 2019, Yang, Qian, et al., 2019).

**FIGURE 2:**
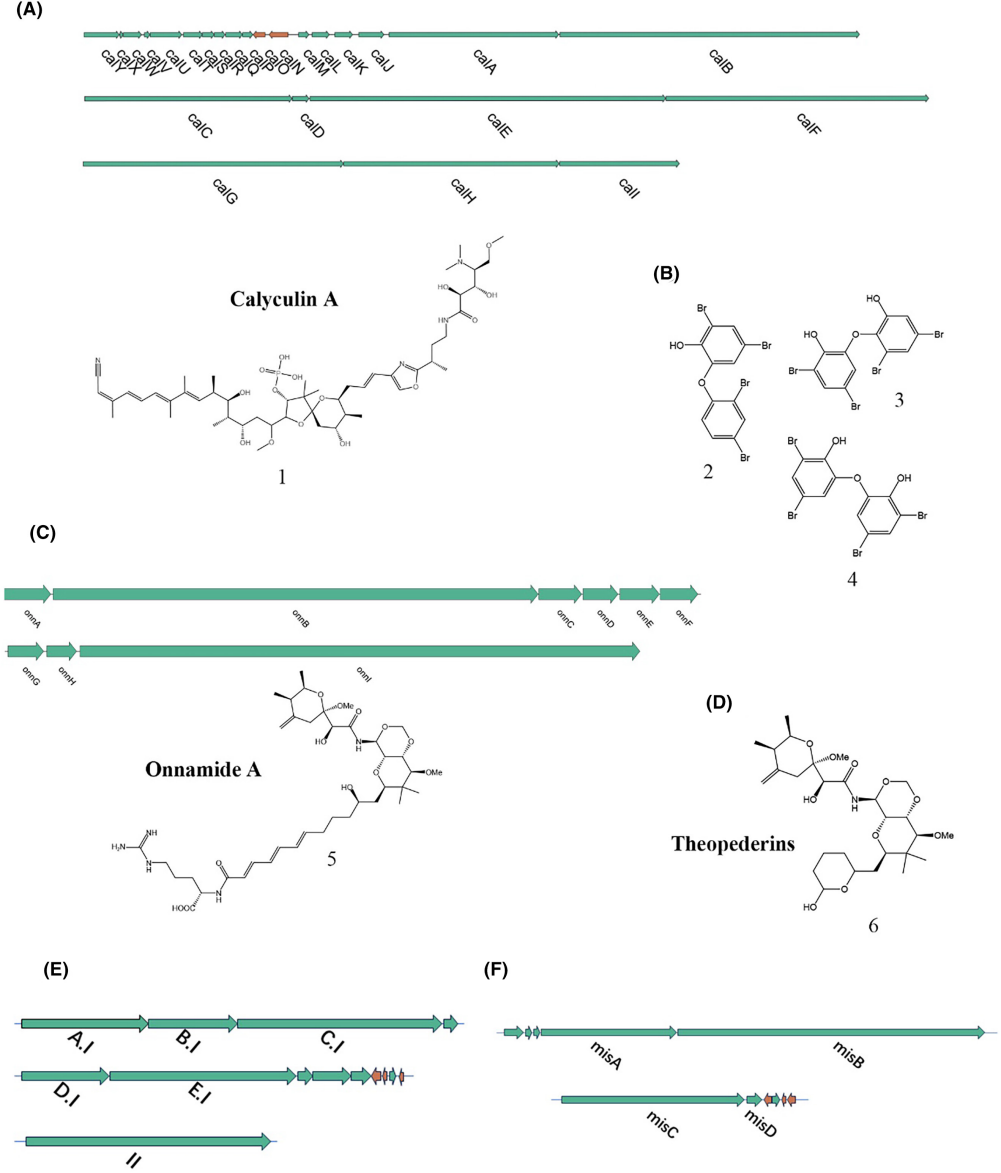
Isolated BGCs and secondary metabolites from marine sponge symbiotic systems. (A) Calyculin A and its BGC detected in the Japanese marine sponge *Discodermia calyx* by metagenome mining and PCR amplification; (B) Various hydroxylated polybrominated diphenyl ethers detected in *Dysideidae* sponges by metagenome mining and PCR amplification; (C) Biosynthesis genes with partial sequences and Onnamide A detected in *Theonella swinhoei* by metagenome mining and PCR amplification; (D) Theopederins A detected in *Theonella swinhoei* by metagenome mining and PCR amplification; (E) Theonellamide BGC detected in Egyptian Red Sea sponge *Theonella* sp. by AntiSMASH; (F) Misakinolide BGC detected in Egyptian Red Sea sponge *Theonella* sp. by AntiSMASH.

Current research on sponge symbiotic system metagenomes predominantly falls into two categories. One approach involves identifying potential BGCs from sponge metagenomes using bioinformatics software like antiSMASH. Subsequent studies focus on these identified BGCs, exploring their biosynthetic capabilities and the secondary metabolites they produce. For instance, Podell and colleagues reconstructed a nearly complete metagenome‐assembled genome of the *Lamellodysidea herbacea*, revealing the secondary metabolite potential of 16 uncharacterized and uncultivated microbial groups associated with this sponge (Podell et al., 2020). Further metagenomic analyses, employing antiSMASH and MIBiG database comparisons, identified unique BGCs in various sponge symbiotic systems. Notably (Dat et al., 2023; Storey et al., 2020), *Acidobacterorita* and *Latescibacteria* symbiotic systems exhibited high biosynthetic potential (Loureiro et al., 2022). These unique BGCs likely contribute to novel synthetic pathways for unknown secondary metabolites. In one example, Nguyen and colleagues discovered a ribosomally synthesized and post‐translationally modified peptide (RiPP) BGC in a marine sponge microbial community. This widely conserved BGC encodes a tryptophan brominase, notable for its independence from prior RiPP core peptide modifications. Shared biosynthetic motifs across different RiPP classes suggest the potential for diverse chemical space in RiPPs, underscoring the potential for discovering new secondary metabolites in sponges (Nguyen et al., 2021). El Samak et al.'s analysis of *Theonella* sp. metagenomics and metatranscriptomics identified numerous actively transcribed BGCs with antimicrobial, cytotoxic, and inhibitory properties. Complete clusters for the biosynthesis of theonellamide (Figure 2E) and misakinolide (Figure 2F) were found, along with clusters closely related to those producing bioactive natural products. The majority of these BGCs were novel, indicating *Theonella* as a key source for bioactive natural product discovery (El Samak et al., 2023).

Beyond the studies mentioned above, metagenomic methods can also be utilized for tracing back to the BGCs and actual producers of known secondary metabolites produced by sponges. Calyculin A (1, Figure 2A), a cytotoxic compound discovered in the Japanese marine sponge *Discodermia calyx* (Ishihara et al., 1989), was studied by researchers who utilized specific primers for PCR amplification of the sponge's metagenomic DNA. This process identified a gene cluster containing trans‐acyltransferase (AT) Type I polyketide synthase (PKS) as a potential BGC for calyculin A. Further analysis confirmed that this gene cluster originated from the symbiotic bacteria *Candidatus* Entotheonell (Wakimoto et al., 2014). This study not only guides the production of calyculin A but also demonstrates the efficacy of metagenomic mining as a powerful tool for isolating biosynthetic genes encoding specific trans‐AT PKS from marine sponge metagenomes. In another study, Agarwal and colleagues utilized metagenomic techniques to identify the hs_bmp BGC, responsible for polybrominated diphenyl ether production in sponges. Following this discovery, they successfully induced expression through vector construction and isolated various hydroxylated polybrominated diphenyl ethers, such as 2,4‐dibromo‐6‐(2,4‐dibromophenoxy)phenol (2), 3,5‐dibromo‐2‐(3,5‐dibromo‐2‐hydroxyphenoxy)phenol (3), and 6,6′‐oxybis(2,4‐dibromophenol) (4, Figure 2B). This research establishes a crucial foundation for further investigation into these compounds. The genetic tools developed for exploring the natural producers of polybrominated diphenyl ethers will facilitate monitoring of the populations responsible for their synthesis. Such advancements hold significant implications for policy formulation and human health (Agarwal et al., 2017). Piel and collaborators made significant discoveries in the metagenome of *Theonella swinhoei*, not only identifying AT PKS genes but also uncovering biosynthetic gene fragments related to onnamides (5, Figure 2C) and theopederins (6, Figure 2D). The genes they isolated encompass nearly the entire region necessary for synthesizing onnamide molecules, which are integral to producing anti‐tumour marine compounds. These gene clusters have a substantial impact on the generation of both known and novel pederin‐type anticancer agents, and their discovery carries important implications for marine biotechnology and ecology (Piel, Hui, Fusetani, & Matsunaga, 2004; Piel, Hui, Wen, et al., 2004).

These specific case studies have significantly broadened our understanding of the diversity of secondary metabolites produced by sponge symbiotic systems. Additionally, they offer essential insights that pave the way for future in‐depth explorations of novel secondary metabolites derived from marine sponges.

### Symbiotic systems of tunicates

Symbiotic systems dominated by tunicates, especially ascidians, have become a focal point in the exploration of secondary metabolites. Much like in sponge symbiotic systems, it is primarily the symbiotic bacteria within tunicates that produce the majority of these secondary metabolites (Chen et al., 2017; Donia et al., 2011; Shenkar & Swalla, 2011).

The research on metagenomes of marine sponges and tunicates has shown remarkable similarities. Tianero and colleagues conducted a study on the metagenomes of 32 different tunicates, revealing species‐specific microbiomes and secondary metabolites, highlighting the vast potential for secondary metabolite mining in tunicate symbiotic systems (Tianero et al., 2015). Similarly, Mohamed S. Donia and his team's research on *Lissoclinum* patella's metagenome led to the isolation of the genome of its symbiotic microbe, *Prochloron didemni*, and examined its biosynthetic capabilities (Donia et al., 2011). This study also underscored the considerable potential for discovering secondary metabolites in tunicate symbiotic systems.

**FIGURE 3:**
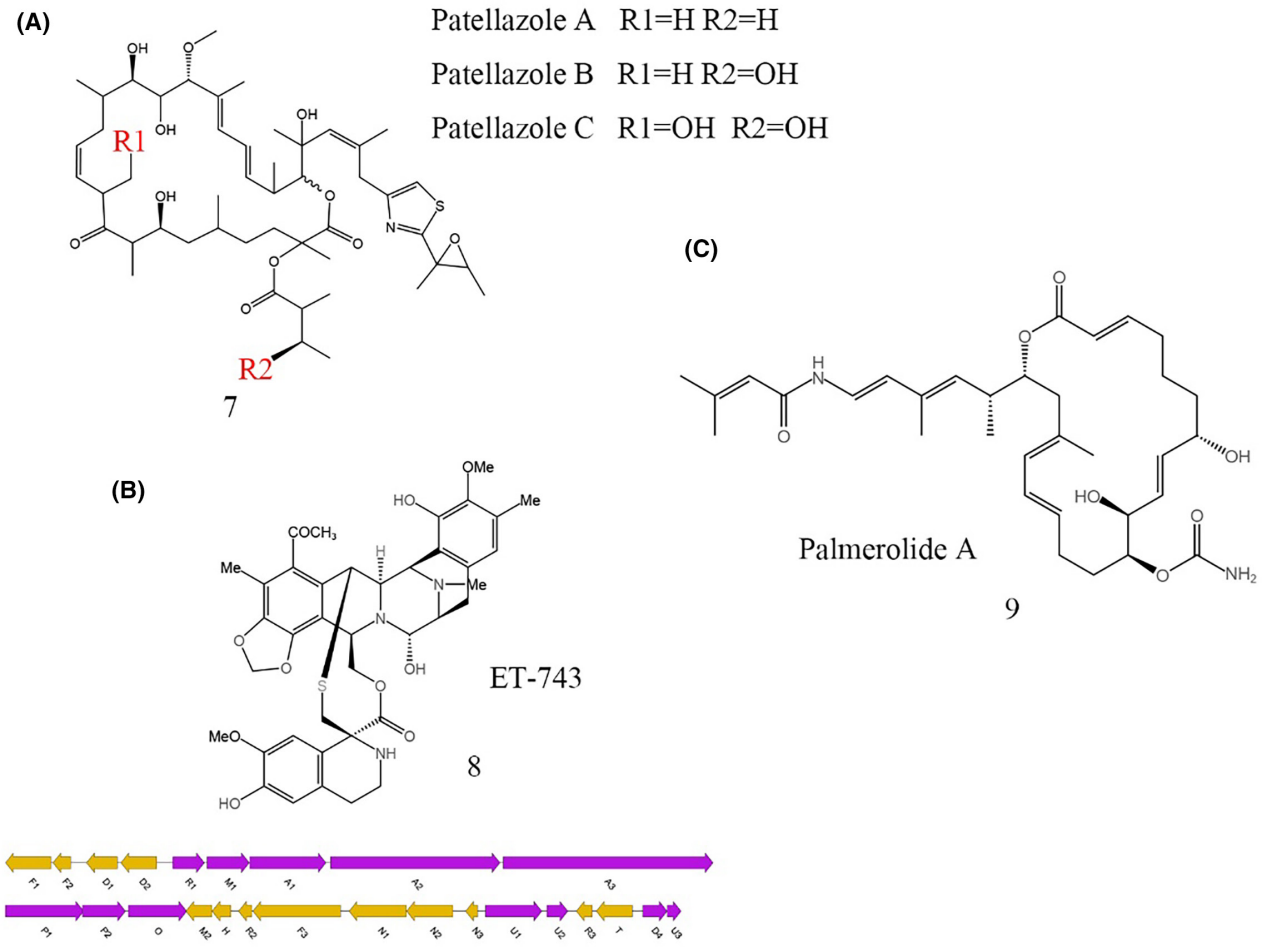
Isolated BGC and secondary metabolites from tunicate symbiotic systems. (A) Patellazoles detected in *Lissoclinum patella* by metagenome mining and PCR amplification; (B) The anticancer agent ET‐743 and its putative BGC isolated from *Ecteinascidia turbinata* by metagenome mining, codon usage bias analysis and metaproteomic analyses; (C) Palmerolide A isolated from *Synoicum adareanum* by metagenome mining and PCR amplification.

In their pursuit of exploring secondary metabolites in tunicates, Kwan and colleagues performed direct sequencing and assembly of the complete genome of *Candidatus Endolissoclinum faulkneri* derived from tunicate metagenomic DNA. Their research led to the identification of the biosynthetic model for patellazoles‐like (7, Figure 3A) compounds. Furthermore, the streamlined genomes of symbiotic bacteria they discovered indicated that secondary metabolism plays a crucial role in symbiotic interactions (Kwan et al., 2012). In another study, Rath and colleagues conducted metagenomic sequencing on the total DNA extracted from a tunicate symbiotic community. They focused on and successfully assembled a continuous 35 kb fragment, which encompassed 25 genes central to the non‐ribosomal peptide synthetase (NRPS) biosynthetic pathway of the ET‐743 anticancer drug. This research confirmed that ET‐743 (8, Figure 3B) is produced by the symbiotic bacterium *Candidatus Endoecteinascidia frumentensis*. Additionally, the study validated the expression of crucial proteins and enzymes involved in this pathway (Rath et al., 2011). In the study conducted by Nicole E. Avalon and colleagues, multiple functional trans‐acyltransferase (AT) domains within the polyketide synthase‐non‐ribosomal peptide synthetase (PKS‐NRPS) structures were identified in the Antarctic ascidian *Synoicum adareanum*. These findings pertain to the inferred BGC for palmerolide A. The research focused on the bioinformatic and mechanistic analysis of the palmerolide PKS‐NRPS biosynthetic pathway, highlighting the intricate relationship between the ascidian's microbiome and its secondary metabolite production (Avalon et al., 2021). Riesenfeld and colleagues successfully identified PKS genes within the metagenome of the tunicate *Synoicum adareanum*'s symbiotic system. Utilizing BLAST analysis for PKS genes, they ascertained that the encoded compound, palmerolide A (9, Figure 3C), recognized for its anti‐melanoma properties, is likely synthesized by symbiotic microorganisms belonging to the γ‐Proteobacteria class (Riesenfeld et al., 2008). Taken together, these studies underscore the crucial role of metagenomics in uncovering secondary metabolites in tunicates and elucidating their biosynthetic origins.

### Other symbiotic systems

Beyond the symbiotic systems of sponges and tunicates, there is notable potential for discovering new secondary metabolites in other symbiotic environments. Lu et al. delved into the metagenomics of microbial communities associated with large algae, predicting a significant number of polysaccharide utilization loci (4451) and BGCs (8810). Their findings revealed that compared to bacteria found in seawater and sediments, those in algal phycospheres possess larger genomes and a relatively higher number of BGCs (Lu et al., 2023). This study underscores the untapped potential of bacteria in algal phycospheres as a rich source for secondary metabolite synthesis.

The aforementioned studies highlight the crucial role of metagenomics in the investigation of secondary metabolites within marine symbiotic systems. By directly obtaining genomic information from environmental samples, metagenomic techniques allow for an in‐depth analysis of complex microbial symbiotic communities. This technology not only reveals the genetic diversity and functional potential of symbiotic microorganisms but also aids in the identification and assembly of BGCs, providing insights into the pathways of secondary metabolite synthesis. Its high‐throughput nature accelerates the discovery of new secondary metabolites, facilitating the efficient screening of potential bioactive molecules on a large scale.

### Challenges and prospects

The exploration of secondary metabolites in marine microorganisms represents a vital research area. The continual discovery and application of various active compounds from these organisms present significant challenges, particularly due to the difficulty of cultivating many of them in pure cultures. Additionally, the high cost and complexity of spectroscopic and mass spectrometric methods for identifying secondary metabolites limit extensive research on new compounds. Therefore, in addressing these practical challenges, especially in the aspect of validation, the use of computational biology tools to mine BGCs of secondary metabolites from metagenomes has become a widely adopted strategy.

The widespread adoption of short‐read sequencing technologies, known for their high accuracy and low cost, has significantly influenced metagenomic research. However, these methods have limitations in genome assembly, particularly in preserving crucial information within the flexible genome where most BGCs are found. This loss of information can significantly impact research on secondary metabolites. In response, there has been a shift towards long‐read sequencing technologies, offering greater throughput and accuracy. For instance, Waschulin and colleagues, for example, recovered over 1400 full‐length BGC sequences from Antarctic soil using third‐generation sequencing (Waschulin et al., 2022). Mantri and team combined long and short reads in a hybrid assembly to study BGCs in the soil of Schönbuch Forest, southern Germany. This approach uncovered many unique BGCs not detectable through short‐read assembly alone (Mantri et al., 2021). Similarly, Sánchez‐Navarro and colleagues observed most contiguous BGCs in activated sludge using long‐read metagenomic data, contrasting with the incomplete results from short‐read data (Sánchez‐Navarro et al., 2022). Huang and team identified 339 BGCs using a smaller set of long‐read sequences, successfully classifying over 89% of them (Huang et al., 2023). These examples underscore the substantial benefits of third‐generation sequencing technologies in preserving comprehensive BGC information.

With the rapid advancement of computer performance and AI, an increasing number of computational biology tools have been developed. In 2021, machine learning methods for BGC prediction and classification underwent significant advancements. GECCO, using Conditional Random Fields (CRF) for BGC prediction, established a more sophisticated benchmark with MIBiG 2.0 (Carroll et al., 2021). Yang et al. enhanced DeepBGC by developing DeepBGCpred, which identified more BGCs by expanding feature dimensions, implementing a sliding window approach, and incorporating negative class categories in Random Forest (RF) classification (Yang et al., 2021). In a similar vein, Liu et al. in 2022 upgraded DeepBGC to e‐DeepBGC, employing comparable strategies to DeepBGCpred, conducting ablation experiments (Liu et al., 2022). In same year, Almeida et al. conducted evaluations of candidate BGCs in two fungal genomes. They employed Q‐learning to refine BGC prediction results, optimizing the prediction process specifically for fungal BGCs (Almeida et al., 2022). In 2023, Rios‐Martinez and team introduced a novel self‐supervised masked language model, primarily utilizing CARP's BiGCARP framework. This model, initially pre‐trained on Pfam‐level using mask LM CARP, was fine‐tuned with a sliding window approach for prediction. Notably, this method achieved, for the first time, the simultaneous detection and classification of BGCs at the Pfam‐level, marking a significant advancement in the field (Rios‐Martinez et al., 2023). The continuous enhancement of algorithms in computational biology tools has led to the identification of more accurate BGCs, aiding in the discovery of novel secondary metabolites. These tools are accessible via the provided web addresses in the accompanying table (Table 1).

**TABLE 1:** Representative artificial intelligence approaches for the exploration of secondary metabolites.

Software	Main methods	Website	References
antiSMASH	pHMM	https://antismash.secondarymetabolites.org/	Blin et al. (2023)
ClusterFinder	HMM	https://github.com/petercim/ClusterFinder	Cimermancic et al. (2014)
DeepBGC	BiLSTM RF	https://github.com/Merck/deepbgc	Hannigan et al. (2019)
GECCO	CRF	https://gecco.embl.de/	Carroll et al. (2021)
Deep‐BGCpred	BiLSTM RF	https://github.com/pmobio/Deep‐BGCpred	Yang et al., 2021)
e‐DeepBGC	BiLSTM RF		Liu et al. (2022)
RLBGC	Q‐learning	https://github.com/bioinfoUQAM/RL‐bgc‐components	Almeida et al. (2022)
BiGCARP	mask LM CARP	https://github.com/microsoft/protein‐sequence‐models	Rios‐Martinez et al. (2023)

The use of computational biology tools has significantly streamlined the identification of potential secondary metabolites in metagenomic data, thereby reducing the complexity of research. However, as deep learning‐based models begin to emerge, the validation of these models heavily relies on comparisons with predictions from traditional models like antiSMASH. Moreover, the claims of discovering new BGCs often lack experimental validation, making it difficult to determine whether these are false positives or genuinely new BGCs. This is a major shortcoming of most AI‐based BGC studies, and we recommend that these studies be more closely linked to practical experiments. Additionally, existing BGC databases have significant issues and are not well‐suited for training more complex models, such as transformers (Vaswani et al., 2017). On one hand, there are too few fully annotated BGC datasets. Current methods, such as DeepBGC, insert known BGCs into unannotated sequences to construct synthetic data, but this synthetic data does not accurately reflect real BGC environments. Furthermore, even these fully annotated BGCs are imbalanced in terms of categories, which is detrimental to data‐driven machine learning. Therefore, in the future, we hope for more comprehensive BGC databases and the development of models that integrate multiple features (as e‐DeepBGC have done) to enhance BGC prediction. Simply transplanting natural language processing techniques does not lead to better performance and interpretability in BGC prediction (Vaswani et al., 2017).

**FIGURE 4:**
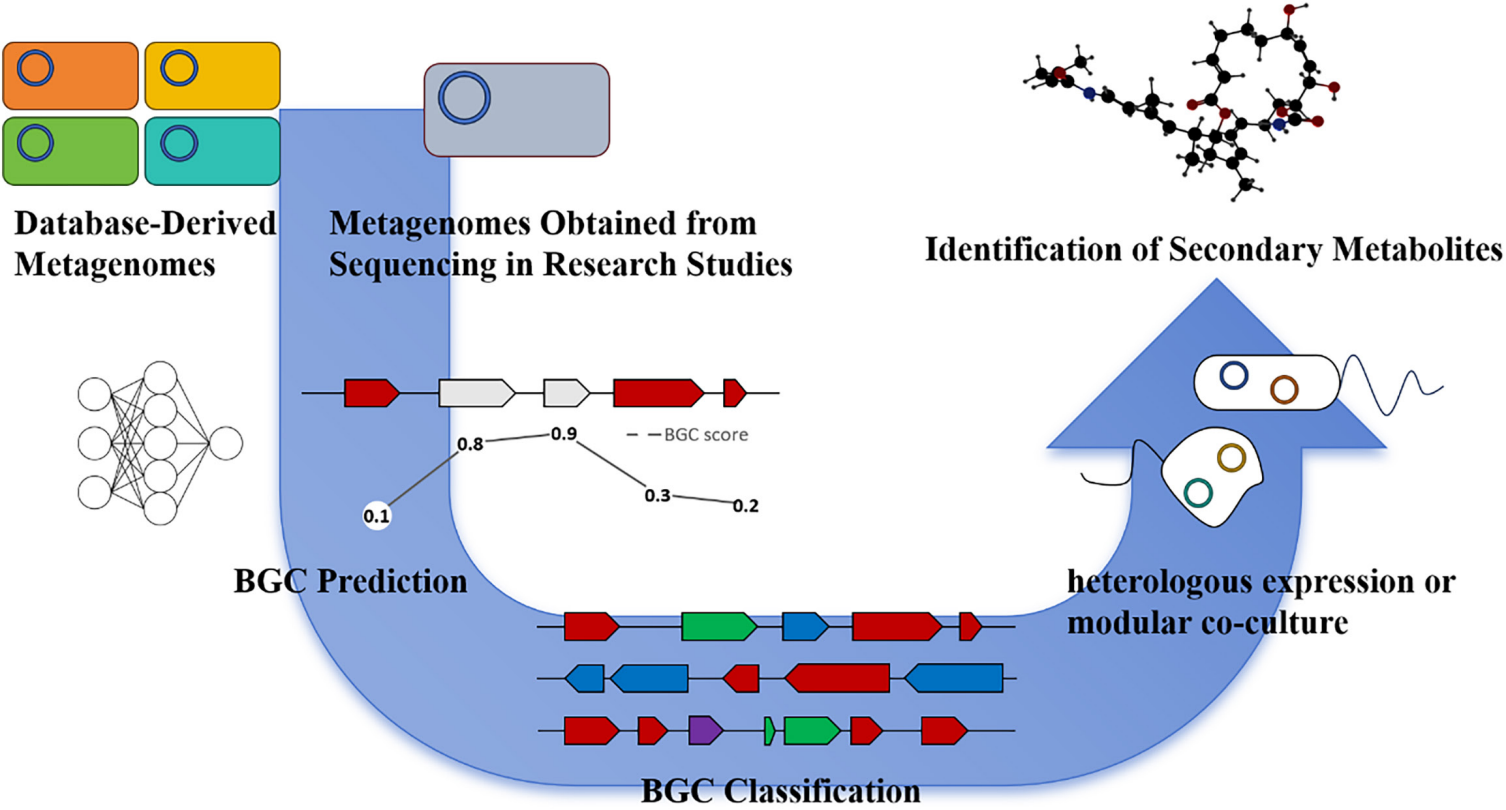
Workflow for predicting and identifying secondary metabolites using metagenomics. Database‐derived metagenomes: Utilization of metagenomic data from existing databases. Metagenomes obtained from sequencing in research studies: Acquisition of metagenomic data from sequencing efforts in various research studies. BGC prediction: prediction of potential BGCs using BGC scoring. BGC classification: classification of predicted BGCs based on their types. Identification of secondary metabolites: detection of secondary metabolites produced by the identified BGCs. Heterologous expression or modular co‐culture: validation of secondary metabolite functions through heterologous expression or modular co‐culture techniques.

BGCs identified in metagenomes through computational biology techniques often correspond to secondary metabolic pathways elusive to pure culture methods. Concurrently, the limitations in heterologous expression technologies pose challenges for accommodating complex BGCs in heterologous hosts, making the practical application and validation of these identified clusters a significant obstacle. The complexity of BGCs, along with transcriptional differences and codon usage variations between *E. coli* and original hosts, often hinders the discovery of new secondary metabolites through metagenomics. Identifying suitable heterologous expression hosts is challenging due to BGC complexity. Even with continuous improvements in *E. coli* expression vectors, new heterologous hosts, and genome engineering, effectively expressing complex BGCs remains difficult. Modular co‐culture engineering, involving complete modularization of biosynthetic pathways with each module regulated in different hosts, emerges as a promising solution. This method, successfully applied in synthesizing natural products from plants and fungi, holds potential for heterologous production of marine secondary metabolites (Trindade et al., 2015; Zhou et al., 2021). Overcoming these challenges could simplify the process from identifying BGCs to elucidating new secondary metabolites (Figure 4). Given the immense potential in discovering secondary metabolites from marine microorganisms and the efficiency of metagenomics in identifying promising BGCs, there is significant growth potential for novel drugs derived from marine microorganisms.

## AUTHOR CONTRIBUTIONS

**Shaoyu Wang:** Writing – original draft; writing – review and editing. **Xinyan Li:** Writing – review and editing. **Weiqin Yang:** Writing – review and editing. **Ranran Huang:** Writing – review and editing.

## CONFLICT OF INTEREST STATEMENT

The authors declare no conflict of interests.
